# NCOA4 requires a [3Fe-4S] to sense and maintain the iron homeostasis

**DOI:** 10.1016/j.jbc.2023.105612

**Published:** 2023-12-28

**Authors:** Hongting Zhao, Yao Lu, Jinghua Zhang, Zichen Sun, Chen Cheng, Yutong Liu, Lin Wu, Meng Zhang, Weijiang He, Shuangying Hao, Kuanyu Li

**Affiliations:** 1 State Key Laboratory of Pharmaceutical Biotechnology, Jiangsu Key Laboratory of Molecular Medicine, Medical School, Nanjing University, Nanjing, China; 2 Department of General Surgery, Nanjing Drum Tower Hospital Clinical College of Nanjing Medical University, Nanjing, China; 3 School of Chemistry and Chemical Engineering, Chemistry and Biomedicine Innovation Center (ChemBIC), Nanjing University, Nanjing, China; 4 School of Medicine, Henan Polytechnic University, Jiaozuo, China

**Keywords:** NCOA4, iron-sulfur protein, protein degradation, ferritin, autophagy, iron metabolism

## Abstract

NCOA4 is a selective cargo receptor for ferritinophagy, the autophagic turnover of ferritin (FTH), a process critical for regulating intracellular iron bioavailability. However, how ferritinophagy flux is controlled through NCOA4 in iron-dependent processes needs to be better understood. Here, we show that the C-terminal FTH-binding domain of NCOA4 harbors a [3Fe-4S]-binding site with a stoichiometry of approximately one labile [3Fe-4S] cluster per NCOA4 monomer. By analyzing the interaction between NCOA4 and HERC2 ubiquitin ligase or NCOA4 and FTH, we demonstrate that NCOA4 regulates ferritinophagy by sensing the intracellular iron-sulfur cluster levels. Under iron-repletion conditions, HERC2 recognizes and recruits holo-NCOA4 as a substrate for polyubiquitination and degradation, favoring ferritin iron storage. Under iron-depletion conditions, NCOA4 exists in the form of apo-protein and binds ferritin to promote the occurrence of ferritinophagy and release iron. Thus, we identify an iron-sulfur cluster [3Fe-4S] as a critical cofactor in determining the fate of NCOA4 in favoring iron storage in ferritin or iron release *via* ferritinophagy and provide a dual mechanism for selective interaction between HERC2 and [3Fe-4S]-NCOA4 for proteasomal degradation or between ferritin and apo-NCOA4 for ferritinophagy in the control of iron homeostasis.

Iron is an essential element involved in fundamental biological processes, including enzymatic catalysis, erythroid generation, mitochondrial respiration, and DNA replication and repair (1). Meanwhile, iron is highly reactive and can be harmful to cells if present in excess (2, 3). Thus, cellular iron homeostasis is subject to strict regulation.

In mammalian cells, iron homeostasis is regulated through the coordination of iron uptake, storage, export, and utilization (4). Ferritin is a vital iron storage protein in cells. It forms a cage consisting of 24 subunits of ferritin heavy chain 1 (FTH) and ferritin light chain, which incorporates up to 4500 iron ions and stores them as mineral cores in its hollow cavity (5). During iron deficiency, ferritin particles are degraded to release iron *via* a process termed ferritinophagy (6, 7). As a selective autophagy receptor, nuclear receptor coactivator 4 (NCOA4) mediates ferritinophagy and promotes iron release from ferritin to maintain the cellular labile iron pool (7). Systemic ablation of *Ncoa4* in murine models leads to the accumulation of tissue ferritin, reduced systemic iron availability, and a functional iron deficiency that manifests as mild hypochromic microcytic anemia (8, 9, 10).

NCOA4-mediated ferritinophagy occurs *via* direct interaction between FTH and NCOA4 that involves a conserved surface arginine (R23) of FTH and a C-terminal domain of NCOA4 (383–522nd) (11). Further studies determined that the amino acids I489 and W497 in the C-terminal structural domain of NCOA4 were important for binding with ferritin. The occurrence of ferritinophagy relies on cellular iron levels. Under iron-repletion conditions, NCOA4 interacts with the HECT and RLD domains-containing E3 ubiquitin protein ligase 2 (HERC2), an E3 ligase, to direct the degradation of NCOA4 through the ubiquitin-proteasome system, thereby inhibiting ferritinophagy and promoting the accumulation of ferritin. This proteasome-mediated process is found to initiate from the iron-binding of NCOA4 (11). Conversely, under iron-depleted conditions, the interaction between NCOA4 and HERC2 is blocked, resulting in increased NCOA4 levels to promote ferritinophagy (11). Very recently, NCOA4 was found to bind iron as an Fe-S cluster, but the cluster type was unclear (12).

By means of integrated biochemical, biophysical, and cellular analyses, we herein report that NCOA4 binds a [3Fe-4S] with conserved cysteine residues, which cluster serves as a critical cofactor for sensing the intracellular iron levels. Under iron-repletion conditions, the ubiquitin ligase HERC2 interacts with NCOA4 dependent on the [3Fe-4S] in NCOA4, dictating the proteasomal degradation of NCOA4. However, when NCOA4 does not bind with [3Fe-4S], it recruits ferritin to mediate the ferritinophagy under iron-depletion conditions.

## Results

### NCOA4 is a [3Fe-4S]-containing protein

**Figure 1:**
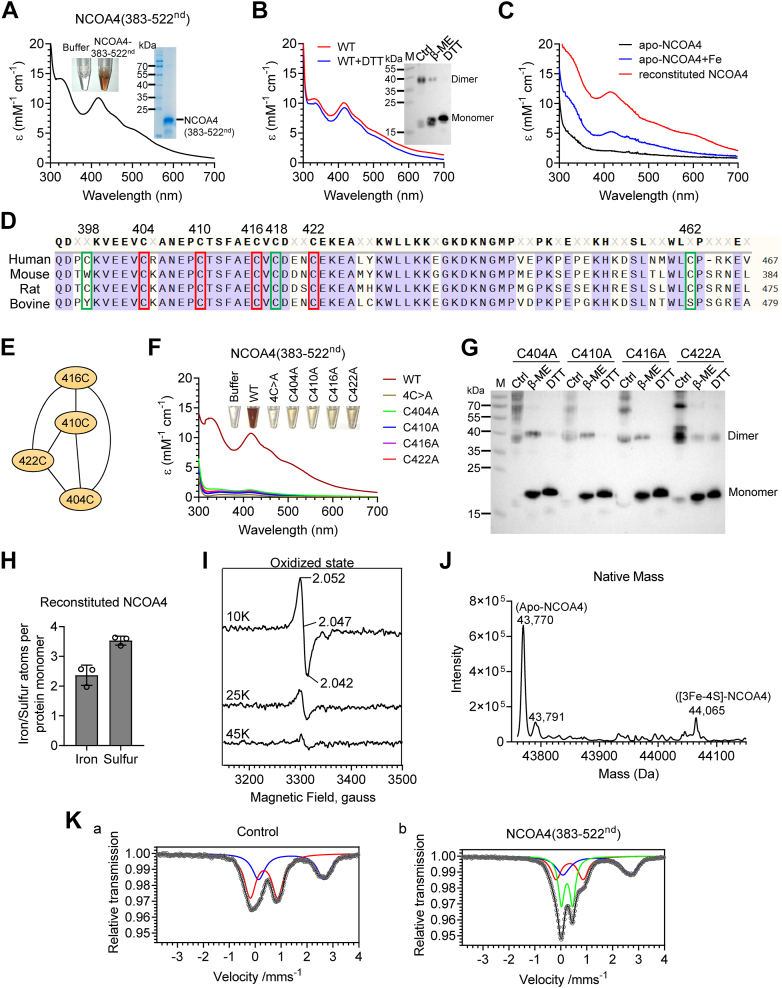
**NCOA4 is a [3Fe-4S]-containing protein.***A*, normalized UV/vis absorption spectra of the purified NCOA4 (383–522nd) protein. The *brownish inset* is freshly purified protein. *Right inset*: Coomassie staining of the purified his-tagged NCOA4 (383–522nd) recombinant protein from *Escherichia coli* in 12.5% SDS-PAGE. The molecular masses (kDa) of the markers are shown. *B*, normalized UV/vis absorption spectra of the purified NCOA4 (383–522nd), treated with/without DTT (5 mM). Inset: Dimerized NCOA4 (383–522nd), determined by nonreducing gel electrophoresis following the treatment with β-ME (10 mM) or DTT (5 mM). *C*, the normalized UV/vis absorption spectra of the apo-NCOA4 (383–522nd), apo-NCOA4 (383–522nd) plus iron, and reconstituted NCOA4 (383–522nd). *D*, sequence alignment of the C-terminal loop region in NCOA4 orthologs from Human (*Homo sapiens*), mouse (*Mus musculus*), rat (*Rattus norvegicus*), and bovine (*Bos taurus*). The strictly conserved residues are colored *purple*. Cysteines suspected of being Fe-S cluster–binding ligands are highlighted in *red*. *E*, the predicted metal-binding sites are shown as a network, which was generated by a pipeline MetalNet (16) based on coevolved metal-binding residues cysteine (C), histidine (H), glutamic acid (E), and aspartic acid (D) (referred as CHED) in the contact map through machine learning. *F*, colors and normalized UV/vis absorption spectra of the purified NCOA4(383–522nd) WT and mutants. *G*, nonreducing gel electrophoresis analysis of mutants treated with β-ME (10 mM) or DTT (5 mM). *H*, chemical estimation of Fe and S content in reconstituted NCOA4 (383–522nd). *I*, continuous-wave EPR spectra recorded on NCOA4 (383–522nd) in the oxidized state. *J*, a native mass assay to show the [3Fe-4S]-bound NCOA4. *K*, Mössbauer spectra of NCOA4. Mössbauer spectra were recorded at T = 80 K with *E. coli* cells carrying either the empty vector pQE80L as a negative control (*a*) or NCOA4(383–522nd) expression plasmid (*b*). The components used for this analysis are described in the text, and the determined Mössbauer parameters are summarized in Table 1. β-ME, β-mercaptoethanol; EPR, electron paramagnetic resonance; NCOA4, nuclear receptor coactivator 4; Fe-S, iron-sulfur.

To determine the form of iron in NCOA4, we constructed and purified the truncated NCOA4 containing amino acids 383 to 522nd, which was demonstrated to contain an iron-binding site (11). Consistent with the iron-binding property of NCOA4, the purified 383 to 522nd NCOA4 (purified NCOA4) was brownish (Fig. 1*A*, see the inset tubes). Its UV/vis absorption spectrum revealed peaks at 320 nm and 416 nm and a broad shoulder at longer wavelengths (Fig. 1*A*), suggesting a [3Fe-4S] (13) or [2Fe-2S] (12, 14). The purified NCOA4 was detected by nonreducing gel and shown to form a stable dimer, in agreement with a previous study (15). DTT or β-mercaptoethanol (β-ME) significantly converted it into monomers, but its color and absorption peaks were not changed (Fig. 1*B*). Therefore, we speculate that the Fe-S cluster might be bound to the NCOA4 monomer and the dimer relies on the disulfide bond between cysteines.

To confirm Fe-S cluster binding in NCOA4, apo-NCOA4 was made by treating purified NCOA4 with potassium ferricyanide and EDTA. UV-Vis spectrum indicated the loss of absorption between 300 to 700 nm (Fig. 1*C*, black line). Incubation of the apo-NCOA4 with iron only under anaerobic conditions for 1 h did not significantly restore the spectral features of the purified NCOA4 (Fig. 1*C*, blue line). However, the Fe-S cluster reconstitution boosted the absorption peaks at 320 and 416 nm (Fig. 1*C*, red line).

Sulfur in cysteines is the most common element to coordinate Fe-S clusters. Sequence alignment showed that human NCOA4 contains seven cysteines in the 383 to 522nd region, including five strictly conserved cysteines Cys404, Cys410, Cys416, Cys418, and Cys422 residues and two unconserved Cys398 and Cys462 (Fig. 1*D*). A software MetalNet (https://github.com/wangchulab/MetalNet) (16) was used to predict the Fe-S binding potential of NCOA4 and Cys404, 410, 416, and 422 residues are the candidates of the coordinated sites (Fig. 1*E*). To validate the Fe-S binding site and the importance of the cysteines, we constructed seven plasmids and purified the mutants of the 383 to 522nd NCOA4 protein with each cysteine residue substitution by alanine. Due to the insolubility of the C398A mutant, we reconstructed another mutant, C398S, which is soluble for characterization. The UV/vis absorption spectra were recorded to see the characteristics of the coordinated Fe-S cluster. As shown in Fig. 1*F*, each single mutation abolished the brownish color or kept a faint color, and the four cysteines mutation 4C > A (all four cysteines replaced by alanine) completely removed the color. The UV/vis absorption peaks at 320 and 416 nm were vanished by each mutation C404A, C410A, C416A, C422A, and 4C > A, indicating the loss of the spectral features of the Fe-S cluster (Fig. 1*F*). The quantification confirmed the loss of iron and sulfide ions within the purified mutants (Fig. S1, *A* and *B*). While the other three NCOA4 mutants, C398S, C418A, and C462A, showed the same color (inset) and UV/vis absorption spectra as WT NCOA4 (Fig. S1*C*), indicating that the three cysteines are not involved in the binding of the Fe-S cluster. These results confirmed the vital role of cysteines in Fe-S coordination.

Cysteine is also a critical residue for protein dimer formation through disulfide bonds. To examine if any cysteine plays a role in the dimerization of NCOA4, we ran the nonreducing gels following the addition of reductant β-ME or DTT. The results showed that the mutation in Cys398, Cys404, Cys410, Cys416, Cys418, Cys422, and Cys462 did not affect the formation of NCOA4 dimer (Figs. 1*G* and S1*D*).

To determine the form of Fe-S cluster binding in NCOA4, the contents of iron and sulfide ions in reconstituted NCOA4 were estimated by ferrozine and methylene blue colorimetric assays, respectively. The results revealed the average association of 2.5 ± 0.28 iron atoms and 3.5 ± 0.12 sulfur per his-tagged NCOA4 monomer (Fig. 1*H*), suggesting a [3Fe-4S] in purified NCOA4. The Fe-S content, the UV-Vis spectrum, and the extinction coefficient of 11,000∼12,580 M^−1^ cm^−1^ at 416 nm indicated the presence of a single [3Fe-4S] cluster in purified NCOA4 (17). To further characterize the iron-sulfur cluster, electron paramagnetic resonance (EPR) was used. The purified NCOA4 was spin-1/2 species, characterized by a rhombic EPR signal with g values at g1  =  2.052, g2  =  2.047, and g3  =  2.042 at 10 K, which signal disappeared at 45 K (Fig. 1*I*), consistent with a [3Fe-4S]^1+^ cluster. Upon dithionite addition, the EPR signal of NCOA4 dissipated (Fig. S2), suggesting a conversion into an EPR silence [3Fe-4S]^0^. To further validate the identity, we subjected the purified GST-NCOA4 (383–522nd) to native protein mass spectrometry analysis. The spectrum exhibited two distinct populations, representing the apo- and the cofactor-bound forms. The mass difference between the two species is 295 Da (Fig. 1*J*), well matching the molecular weight of a [3Fe-4S].

To further verify the coordination of a [3Fe-4S]^1+^ by NCOA4, supplementary zero-field ^57^Fe Mössbauer spectroscopic measurements were performed with NCOA4(383–522nd) overexpressed *Escherichia coli*. The Mössbauer spectrum was recorded at T = 80 K and compared with nonrecombinant *E. coli* as a negative control (Fig. 1*K* and Table 1). The Mössbauer spectrum in control fitted well with two individual components (Fig. 1*Ka*, lines blue and red). However, the NCOA4-expressed sample exhibited an additional component (Fig. 1*Kb*, line green) with an isomer shift of δ = 0.24 mm s^−1^ and quadrupole splitting of ΔE_Q_ = 0.43 mm s^−1^ (Table 1) representing ∼36% of the overall iron (^57^Fe) content (Fig. 1*Kb*). These δ and ΔE_Q_ values are in agreement with those found for other cuboidal [3Fe-4S] clusters in the 1+ oxidation state (18), corroborating the results of our EPR spectroscopic studies. Collectively, our results demonstrated that the purified NCOA4 is a [3Fe-4S] protein.

**Table 1:** Mössbauer parameters obtained from the fit of Mössbauer spectra recorded at T = 80 K for nonproducing *Escherichia coli* cells as control (a) and for the identical strain producing NCOA4 (383–522nd) (b)

Component	*δ* (mm s^−1^)	Δ*E*_Q_ (mm s^−1^)	*Γ*_HWHM_ (mm s^−1^)	Relative contribution (%)
a. Control				
Red line				
Ferric high-spin, Fe^3+^	0.34	1.06	0.52	60
Blue line				
Ferrous high-spin, Fe^2+^	1.39	2.52	0.62	40
b. NCOA4(383–522nd)				
Red line				
Ferric high-spin, Fe^3+^	0.34	1.02	0.47	32
Blue line				
Ferrous high-spin, Fe^2+^	1.36	2.58	0.64	32
Green line				
[3Fe-4S]^1+^	0.24	0.43	0.27	36

NCOA4, nuclear receptor coactivator 4.

### The HERC2 interacts with a Fe-S binding NCOA4 for NCOA4 proteosome-dependent degradation

**Figure 2:**
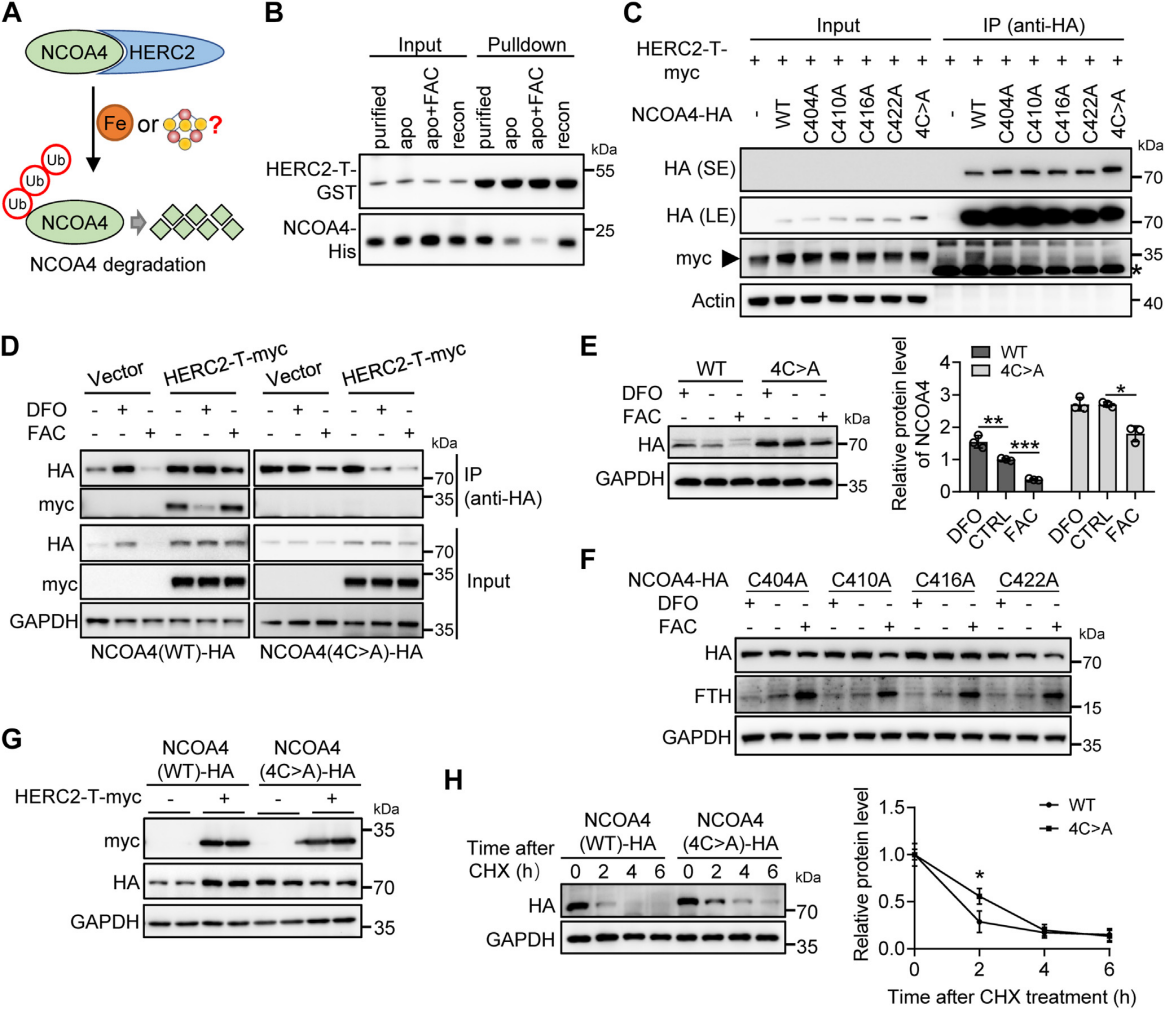
**The NCOA4–HERC2 interaction relies on the Fe-S cluster.***A*, a schematic mode of interaction between NCOA4 and HERC2. *B*, direct interaction between purified, apo-, or reconstituted (recon) NCOA4 (383–522nd) and HERC2, detected by GST pull-down assays. *C*, the interaction between NCOA4 and HERC2 was abolished by cysteine mutation. HEK293 cells were transiently cotransfected to express HA-tagged NCOA4 (WT) or mutants and myc-tagged HERC2 (2540–2700th). Post transfection 24 h, cells were harvested and lysed. Whole-cell extracts were subjected to immunoprecipitation (IP) using anti-HA magnetic beads and immunoblotting with the antibodies against HA and myc. -: an empty plasmid pcDNA3.1(−). The *asterisk on the right* is the conjugated light chain of the second antibody. *D*, the interaction between NCOA4 (WT) and HERC2 was regulated by iron treatment. HA-tagged NCOA4 (WT) or mutants and myc-tagged HERC2 (2540–2700th) were coexpressed in HEK293 cells, followed by DFO (100 μM) or FAC (200 μM) treatment for 6 h and IP was performed as in (*C*). *E*–*F*, the WT and mutant protein levels of NCOA4 in response to iron treatment. HEK293 cells were transfected to express NCOA4 (WT) or mutant, followed by the addition of FAC (200 μM) or DFO (100 μM) and incubation for 6 h. Then, immunoblot analysis was performed with antibodies against HA, FTH, and GAPDH. n = 3. ∗*p* < 0.05, ∗∗*p* < 0.01, and ∗∗∗*p* < 0.001. *G*, HERC2 (2540–2700th), a CUL7 domain without ubiquitinating activity, stabilized the exogenous NCOA4 in an Fe-S cluster–dependent manner. HA-NCOA4 WT or mutants and myc-HERC2 were coexpressed in HEK293 cells for 24 h and subjected to SDS-PAGE and immunoblot to detect exogenous NCOA4 abundance. *H*, the increased turnover rate of NCOA4 mutants. HEK293 cells were transfected to express NCOA4 (WT) or mutant for 24 h, followed by exposure to cycloheximide (100 μg/ml) for the indicated times. The immunoblot analysis was performed. The band intensity of NCOA4 (WT)-HA and NCOA4 (4C > A)-HA was quantitated by ImageJ (https://imagej.net/software/imagej/) analysis. n = 3. ∗∗*p* < 0.01. DFO, deferoxamine; FAC, ferric ammonium citrate; Fe-S, iron-sulfur; HA, hemagglutinin; HEK, human embryonic kidney; NCOA4, nuclear receptor coactivator 4.

To reveal the rationale for the Fe-S binding of NCOA4, we examined whether the interaction between NCOA4 and HERC2 ligase is Fe-S cluster-dependent. HERC2 selectively recognizes NCOA4 under high iron conditions to mediate NCOA4 turnover *via* the ubiquitin-proteasome system, thereby reducing NCOA4 levels and maintaining high levels of ferritin (11), illustrated in Figure 2*A*. We then freshly prepared NCOA4, apo-NCOA4 (after removal of Fe-S), and reconstituted NCOA4. GST pull-down confirmed that HERC2 preferred binding with Fe-S-bound NCOA4 rather than with apo-NCOA4. Iron addition did not rescue the interaction of apo-NCOA4 with HERC2 (Fig. 2*B*), supporting the Fe-S cluster dependence in the interaction. To further confirm the scenario within cells, we transfected human embryonic kidney (HEK293) cells to express the myc-tagged truncated HERC2 (amino acids 2540–2700th, abbreviated as HERC2-T) and HA (hemagglutinin)-tagged full-length NCOA4. HERC2-T contains an NCOA4-binding domain, not a domain for ubiquitination to execute NCOA4 proteasomal degradation (11, 19). By coimmunoprecipitation assays, we showed that, unlike the WT NCOA4, the mutants all were unable to bind HERC2 (Fig. 2*C*), suggesting the crucial role of the Fe-S cluster in the interaction between NCOA4 and HERC2. Taking the concept that Fe-S cluster biogenesis is expected to be enhanced under high iron conditions, we next evaluated the effects of the mutation on their interaction under different iron conditions. The results showed that the interaction between WT NCOA4 and HERC2-T was weakened under iron depletion conditions and strengthened under iron-repletion conditions (Fig. 2*D*, left panel). However, NCOA4 mutant 4C > A, unable to coordinate Fe-S cluster, could not bind to HERC2-T, regardless of intracellular iron status (Fig. 2*D*, right panel). These results prove that the integrity of the Fe-S cluster is essential for the NCOA4–HERC2 interaction.

Iron availability regulates Fe-S cluster biogenesis. With more iron within a broad concentration, more Fe-S clusters are assembled for many Fe-S proteins or enzymes involved in various bio-processes. Interestingly, when expressing either HA-tagged full-length WT NCOA4 or mutants, singles or 4C > A, in HEK293 cells, we found that the WT, not the mutants, was well regulated by iron status, that is, downregulated by iron repletion and upregulated by iron depletion (Fig. 2, *E* and *F*). This difference implies that the Fe-S cluster in NCOA4 is a regulatory prosthetic group that renders NCOA4 stability. Even under normal iron conditions, the immunoblotting showed higher protein levels of mutant than WT (lanes 2 and 5 in Fig. 2*E* and lanes 1/2 and 5/6 in 2G), suggesting that the Fe-S cluster-bound NCOA4 interacts with endogenous HERC2 for rapid proteasomal degradation. Then, we asked what if the interaction was competitively blocked by simultaneously expressing myc-tagged HERC2-T. The immunoblot analysis revealed a greater abundance of HA-tagged WT NCOA4 with HERC2-T expression than without (Fig. 2*G*, lanes 3/4 *versus* lanes 1/2). However, this effect was not observed when coexpression of HERC2-T with NCOA4 mutant 4C > A (Fig. 2*G*, lanes 5/6 *versus* lanes 7/8), suggesting that Fe-S cluster binding unfavors the stability of NCOA4. Then, the WT and mutant turnover was measured by cycloheximide chase analysis. We found that the half-life of the NCOA4 mutant 4C > A in HEK293 cells was substantially longer than that of the WT protein (Fig. 2*H*).

### A deficiency of Fe-S cluster biogenesis enhances the stability of NCOA4

**Figure 3:**
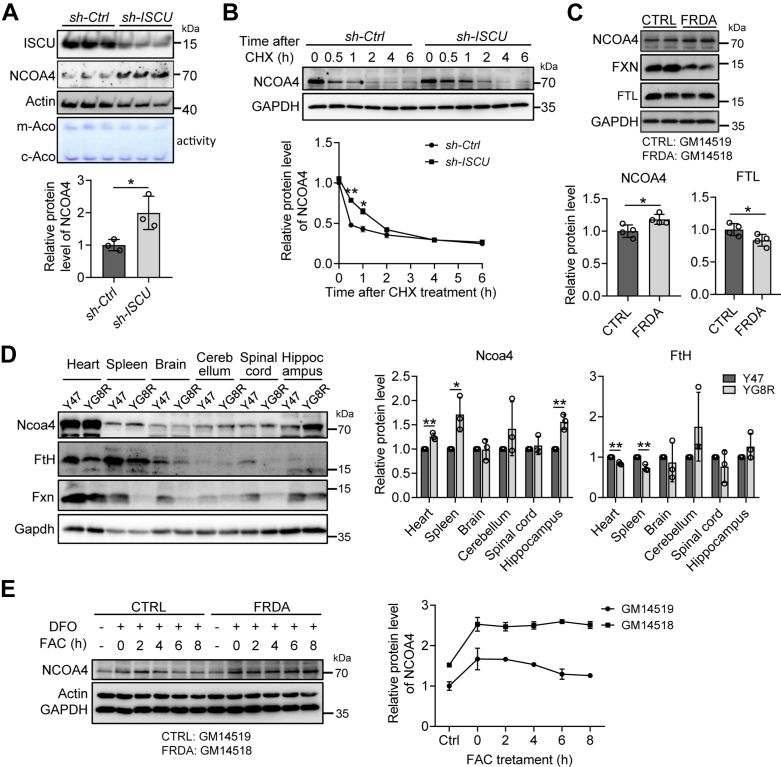
**Loss of Fe-S cluster biogenesis enhances the stability of NCOA4.***A*, NCOA4 protein levels after knockdown of *ISCU*, encoding a scaffold protein for Fe-S biogenesis. The shRNA-mediated (target sequence of *ISCU*: ATTGTGGATGCTAGGTTTA) knockdown in HEK293 cells was performed for 48 h, followed by immunoblot analysis or in-gel aconitase assay (labeled by activity). The bottom is the quantified NCOA4 protein levels. n = 3. ∗*p* < 0.05. *B*, the prolonged turnover rate of NCOA4 post knockdown of *ISCU*. HEK293 cells were transfected as in (*A*) and then exposed to cycloheximide (100 μg/ml) for the indicated times before immunoblot analysis. The band intensity was quantitated by ImageJ software. n = 3. ∗*p* < 0.05, and ∗∗*p* < 0.01. *C*, NCOA4 expression levels in FXN-deficient cells, revealed by immunoblot analysis. GM14519: derived from a healthy control; GM14518: derived from a Friedreich ataxia (FRDA) patient. n = 4. ∗*p* < 0.05. *D*, Ncoa4 protein levels in YG8R mice, a FRDA mouse model, detected by immunoblot analysis. Y47: control mice *versus* YG8R. *Bottom panel:* the quantification of Ncoa4 protein levels. n = 3. ∗*p* < 0.05 and ∗∗*p* < 0.01. *E*, NCOA4 protein levels in GM14519 (CTRL) and GM14518 cells (FRDA) in response to iron treatment. The cells were treated with 100 μM DFO for 8 h, followed by 200 μM FAC for the indicated hours. Whole-cell lysates were analyzed by immunoblotting. n = 3. DFO, deferoxamine; FAC, ferric ammonium citrate; Fe-S, iron-sulfur; FXN, frataxin; HEK, human embryonic kidney; NCOA4, nuclear receptor coactivator 4.

Next, we evaluated the stability of NCOA4 when Fe-S cluster biogenesis was disturbed. Knockdown of a gene *ISCU*, encoding a scaffold protein for Fe-S cluster biogenesis, was performed in HEK293 cells by shRNA. *ISCU* was successfully knocked down, and the consequence was approved by the decrease in the activity of mitochondrial aconitase (m-Aco) as shown with in-gel assays (Fig. 3*A*). In agreement with data in Figure 2, *D*–*H*, endogenous NCOA4 was increased significantly after *ISCU* knockdown (Fig. 3*A*). Moreover, cycloheximide chase analysis revealed that the turnover rate of the NCOA4 was substantially longer after *ISCU* knockdown (Fig. 3*B*), in agreement with the suggestion that Fe-S binding regulates NCOA4 stability (12).

To further verify the importance of the Fe-S cluster in NCOA4 turnover, we used other Fe-S cluster deficiency models: lymphocytes derived from Friedreich’s ataxia (FRDA) patient and YG8R mice with frataxin (FXN) deficiency (20, 21). FXN, as an allosteric activator, plays an important role in Fe-S cluster biogenesis in eukaryotic organisms (22, 23, 24). Then, we asked whether an increase in NCOA4 could be observed and whether ferritinophagy was promoted in cells GM14518 derived from an FRDA patient compared with the cells GM14519 from the patient’s healthy mother. The results showed a significant increase of NCOA4 protein levels in GM14518 compared to GM14519, which correlated with the decreased ferritin (Fig. 3*C*). Similarly, NCOA4 was significantly upregulated in a variety of tissues, such as the heart, spleen, and hippocampus, in YG8R mice of FRDA model (Fig. 3*D*), proving that the absence of Fe-S clusters promotes the stability of NCOA4. Iron supplementation has partially restored Fe-S cluster synthesis in FRDA cell models (25). Then, we examined the changes of NCOA4 following the transition from low iron to high iron conditions. The results showed that NCOA4 levels decreased gradually in GM14519 cells but kept constant in GM14518 cells upon iron repletion (Fig. 3*E*, lanes 0–8 h), further proving the vital role of the Fe-S cluster in the stability of NCOA4.

### Apo-NCOA4, not Fe-S–containing NCOA4, interacts with ferritin to mediate ferritinophagy and regulates cellular iron homeostasis

**Figure 4:**
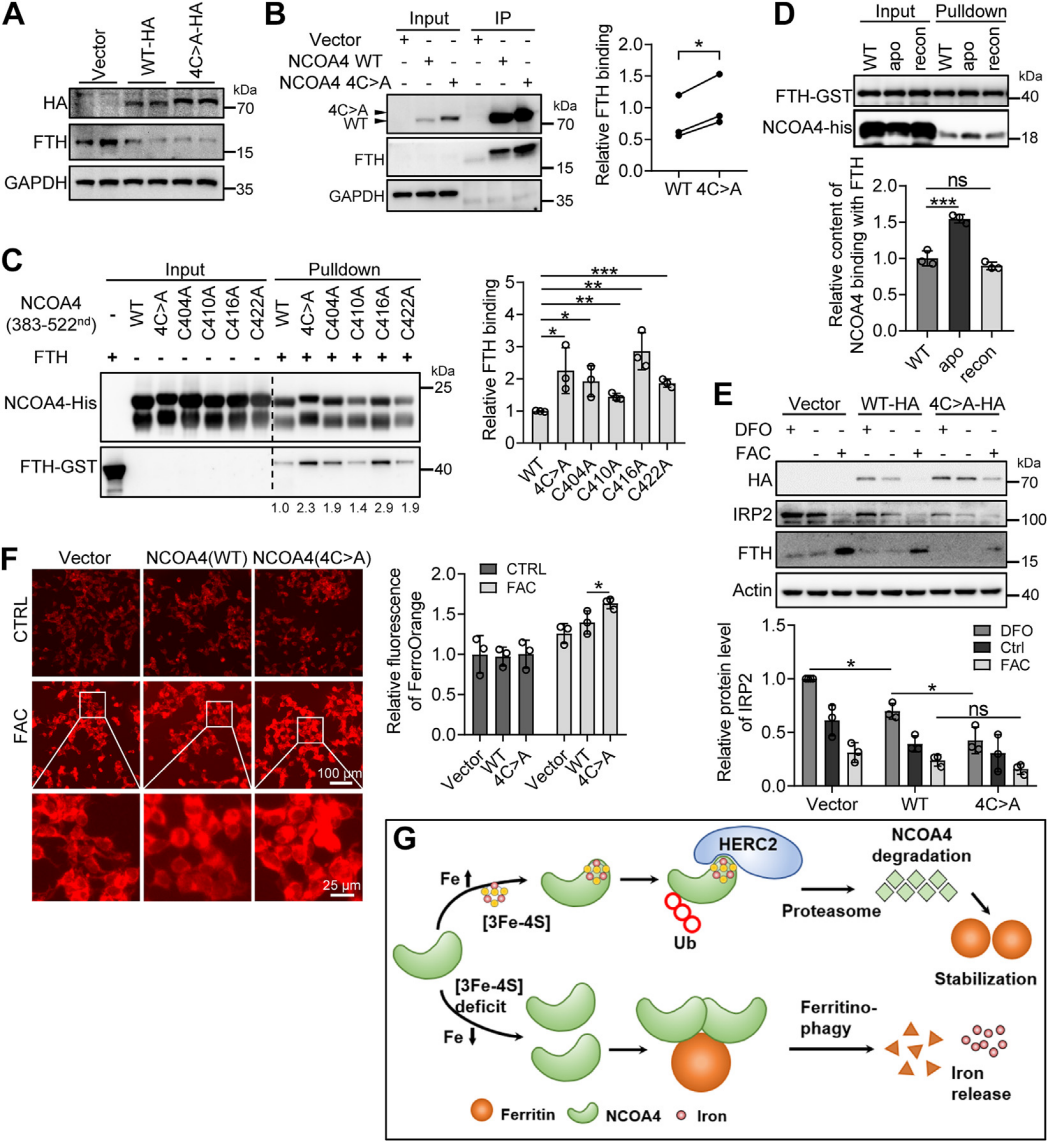
**NCOA4 interacts with ferritin in a Fe-S cluster–free manner to regulate iron homeostasis.***A*, ferritin (FTH) levels in overexpressed NCOA4 WT and mutant cells. HEK293 cells were transiently transfected to express HA-tagged NCOA4 WT or mutant (4C > A). After transfection 24 h, cells were harvested and lysed for immunoblotting. GAPDH was used as an internal control. Vector: empty vector pcDNA3.1(−). *B*, a stronger interaction between FTH and NCOA4 mutant than between FTH and WT. Transfection was the same as in (*A*). Whole-cell extracts were subjected to immunoprecipitation (IP) using anti-HA magnetic beads and immunoblotting with antibodies against HA, FTH, and GAPDH. n = 3. ∗*p* < 0.05. *C*, direct interaction between NCOA4 (383–522nd) WT or mutants and FTH, detected by His-pulldown assays. His-tagged NCOA4 (383–522nd) and GST-tagged FTH were expressed in *Escherichia coli* and purified, respectively. n = 3. ∗*p* < 0.05, ∗∗*p* < 0.01, and ∗∗∗*p* < 0.001. *D*, direct interaction between apo- or reconstituted (recon)- NCOA4 (383–522nd) and FTH, detected by GST pull-down assays. Protein expression and purification are the same as in (*C*). His/GST pulldown and apo-NCOA4 and reconstituted-NCOA4 preparation see Materials and methods. *E–F*, loss of Fe-S cluster in NCOA4 disturbs iron homeostasis. HEK293 cells were transiently transfected to express HA-tagged NCOA4 WT or mutant (4C > A). After transfection 24 h, cells were treated with DFO (100 μM) or FAC (200 μM) for 12 h. Whole-cell extracts were subjected to immunoblotting against HA, IRP2, FTH, and actin (*E*) or intracellular Fe^2+^ was probed by FerroOrange, imaged with a fluorescence microscope (*F*). n = 3. ∗*p* < 0.05. *G*, regulation model for the HERC2–NCOA4–FTH axis by iron and Fe-S cluster. Under iron-repletion conditions, the Fe-S cluster is incorporated into the C-terminal domain of NCOA4 to form a holo-NCOA4. HERC2 recognizes and recruits holo-NCOA4 as a substrate for polyubiquitination and degradation, favoring ferritin iron storage. On the contrary, iron depletion disadvantages Fe-S biogenesis, resulting in NCOA4 apo-protein, favoring ferritin binding to promote ferritinophagy and release iron. DFO, deferoxamine; FAC, ferric ammonium citrate; Fe-S, iron-sulfur; HA, hemagglutinin; HEK, human embryonic kidney; IRP2, iron regulatory protein 2; NCOA4, nuclear receptor coactivator 4.

NCOA4 is a selective cargo receptor that mediates the autophagic degradation of ferritin; thus, it is crucial for iron homeostasis (6, 7). We have demonstrated the importance of Fe-S in the interaction between NCOA4 and HERC2 under iron-repletion conditions. Then, we wonder if NCOA4 interacts with FTH in an iron-free manner under iron transition-to-depletion conditions to proceed with the NCOA4-mediated ferritinophagy. To confirm the hypothesis, WT and mutant 4C > A NCOA4 were expressed in HEK293 cells, and the endogenous FTH was detected. The results showed that FTH significantly decreased regardless of the WT or mutant expression (Fig. 4*A*), suggesting that both can mediate ferritinophagy. Does FTH prefer binding apo-NCOA4 to promote ferritinophagy? To answer this question, we examined the binding of endogenous FTH to the exogenously expressed NCOA4 WT and mutant. Through coimmunoprecipitation, we found that the mutant showed a more robust capability to bind FTH than the WT did (Fig. 4*B*). To examine the direct interaction between them, we purified the GST-tagged FTH and his-tagged NCOA4 WT or mutant and compared the interaction between them by His-pulldown assays. Each mutation, either single cysteine substitution or four cysteine residues substitution by alanine, pronouncedly enhanced the ability of NCOA4 to interact with FTH, revealed by immunoblotting (Fig. 4*C*). To further validate the importance of the Fe-S cluster coordination with the reconstituted NCOA4 in vanishing the interaction, the freshly prepared NCOA4, apo-NCOA4, and reconstituted NCOA4 were used. GST pull-down confirmed that FTH preferred binding with apo-NCOA4 rather than Fe-S-loaded NCOA4 (Fig. 4*D*).

Then, we wonder if NCOA4 senses the cellular iron status through Fe-S binding to modulate iron homeostasis. Due to the failure to construct an NCOA4 KO mutant due to its indispensability, we overexpressed NCOA4 WT and mutant, followed by iron addition or depletion. Given that the overexpressed NCOA4 mutant pushed ferritinophagy by interaction with FTH, we examined the iron status by detecting iron regulatory protein 2 (IRP2), an iron sensor. We found that IRP2 levels reduced more significantly after NCOA4 mutant expression than WT expression under iron depletion conditions (Fig. 4*E*, lanes 1, 4, and 7). In contrast, no significant change was observed under high iron conditions (Fig. 4*E*, lanes 3, 6, and 9). Accordingly, the more stable the NCOA4 is, the less ferritin is (Fig. 4*E*, lanes 4 and 7 under iron depletion conditions, lanes 6 and 9 under iron-repletion conditions). The low levels of ferritin under iron-repletion conditions suggest massive iron in the labile iron pool, which was verified by the FerroOrange probe (Fig. 4*F*). Therefore, we conclude that apo-NCOA4 recruits ferritin to mediate ferritinophagy under iron-depletion or deficiency of Fe-S cluster conditions to modulate cellular iron homeostasis.

## Discussion

In this study, we uncover a [3Fe-4S] as a NCOA4 cofactor, which is critical in controlling the abundance of NCOA4 and ferritinophagy to maintain iron homeostasis. In combination with the previous studies on iron-dependent NCOA4 turnover to control ferritinophagy (11), our findings unveil a plausible mechanism by which NCOA4 senses the levels of iron through its Fe-S to dictate iron storage or iron release. We propose that the Fe-S cluster, whose biogenesis is facilitated under iron-sufficient conditions, is incorporated into the C-terminal domain of NCOA4 to form a [3Fe-4S]–NCOA4 complex. HERC2 recognizes and recruits [3Fe-4S]-NCOA4 as a substrate for polyubiquitination and degradation, favoring ferritin iron storage. On the contrary, iron depletion disadvantages Fe-S biogenesis, resulting in NCOA4 in apo-form interacting with ferritin for ferritinophagy and iron release (Fig. 4*G*).

The stoichiometric ratio of the NCOA4 and [3Fe-4S] as a complex is ascertained by biochemical assays in this study to be 1:1. The cysteines C404A, C410A, C416A, and C422A are predicted for the iron-sulfur coordination. We confirmed the importance of these cysteines, but we have yet to figure out which one cysteine is essential for [3Fe-4S] coordination, but not a direct ligand since each four cysteines mutation drastically abolishes the [3Fe-4S] integrity. An Autodock software ((26) version 4.2.6, the Scripps Research Institute, https://autodock.scripps.edu/download-autodock4/) was used with a retrieved structure from AlphaFold (code: AF-Q13772-F1). The docking simulation favors a [3Fe-4S] in a specific loop region, close to C404, C410, C416, and C422 (5–6.6 Å, Fig. S3). Overall, the distance is farther here than other reported Fe-S protein (27) between iron and cysteine sulfur. It is very likely that the conformation changes when Fe-S inserts for better coordination. The crystallographic structural approaches are required to determine what the NCOA4-[3Fe-4S] complex looks like.

Fe-S clusters are essential cofactors required throughout the clades of biology, such as DNA repair, mitochondrial respiration, and erythroid differentiation (28). In mammals, a few proteins have been demonstrated to sense cellular iron levels by gain or loss of the Fe-S cluster. The first example is IRP1, which functions either as an active [4Fe-4S] cytosolic aconitase in iron-repletion cells or as an apo-protein to bind an iron-responsive element present in the 5′- or 3′- UTR of mRNA in iron-depletion cells (29). The second one is FBXL5, which was recently uncovered with a [2Fe-2S] cofactor playing a critical role in controlling the recruitment and polyubiquitination of IRP2 in iron homeostasis (27). The data from this study solidify the role of [3Fe-4S] for NCOA4 as another iron-sensing protein to control cellular iron homeostasis. In agreement with these, increased IRP2 and decreased ferritin were observed in FXN-deficient cells derived from FRDA patients (25), which may be the outcome of the insufficient Fe-S supply to both FBXL5 and NCOA4, supporting the previous studies (12, 25, 30). Interestingly, the effects of NCOA4 on protein levels of IRP2 suggest that overexpressed cellular NCOA4 might not be all in Fe-S–binding form under iron-sufficient conditions and not all in Fe-S–free form under iron deprivation conditions. This explains the reduced IRP2 when NCOA4 is overexpressed under different iron conditions.

NCOA4 is regulated in various ways and cell type–specific. At the transcriptional level, HIF2α promotes the transcript of NCOA4 under hypoxia or iron deficiency circumstances to impact iron homeostasis in the intestine and liver (10, 31). Another study has demonstrated that a significant transcriptional downregulation of NCOA4 was observed in macrophages or BMDM after hypoxic stimulation (32). However, hypoxia rescues frataxin loss by restoring Fe-S cluster biogenesis (33), which would promote HERC2-dependent degradation of NCOA4 due to Fe-S cluster acquisition. Our previous study revealed that HIF2α was significantly upregulated in *Irp2*-deficient mice (34, 35), accompanied by decreased NCOA4 protein levels and weakened ferritinophagy in the gut and liver (36). Stimulating ferritinophagy by HIF2 inhibition facilitates iron release from ferritin to improve anemia (36). These studies all indicate that the participation of NCOA4 in ferritinophagy may also be regulated by oxygen at transcriptional and/or posttranslational levels in a context dependence of physiological conditions, which has recently been revealed (12).

In summary, we characterized the [3Fe-4S]-binding property of NCOA4 and identified the vital cysteines for [3Fe-4S] coordination. We also revealed the physiological significance of the [3Fe-4S] binding. When intracellular Fe-S clusters are deficient, NCOA4 is liberated from the grasp of HERC2 and its stability is improved, which in turn mediates ferritinophagy and iron release. On the contrary, NCOA4 acquires Fe-S cluster for HERC2-mediated proteasomal degradation and for improvement of ferritin stability and iron storage. Thus, inadequate biogenesis of Fe-S clusters likely also suffers iron toxicity resulting from the uncontrolled ferritinophagic flux as in FRDA.

## Experimental procedures

### Mammalian cell culture

Cells were cultured in a tissue culture incubator at 37 °C and 5% CO_2_. HEK293/HEK293T were purchased from the American Tissue Culture Collection and tested negative for *mycoplasma* contamination using a Universal *Mycoplasma* Detection kit from American Tissue Culture Collection. These cell lines were maintained in Dulbecco’s modified Eagle’s medium (GIBCO) supplemented with 10% fetal bovine serum and 1% penicillin/streptomycin. Human healthy control and matched FRDA patients–derived lymphoblasts were purchased from the Coriell Institute for Medical Research repository. The cell lines used are GM14519 and GM14518, free from *mycoplasma* contamination. Cells were cultured in medium RPMI 1640 (Solarbio Science & Technology Co Ltd) with 10% fetal bovine serum and 1% penicillin/streptomycin.

### Animals and tissue collection

Y47 and YG8R mice were purchased from Jackson Laboratory. Animals were group-housed under standard housing conditions with a 12-h light-dark cycle and temperature of 25 °C. All animal experiments were reviewed and approved by the Animal Investigation Ethics Committee of Nanjing University and were performed according to the Guidelines for the Care and Use of Laboratory Animals published by the National Institutes of Health.

### Plasmid constructs

The constructed plasmids are listed in Table S1. The human *NCOA4α* variant (encoding amino acids 1–614, NM_001145263.1, WT, or point mutant) lacking the stop codon was cloned into pcDNA3.1(−)-HA. The truncated fragment of *NCOA4* was cloned into pQE80L to express the 383 to 522nd NCOA4 in *E. coli*. Site-directed mutagenesis was performed using the Beyotime QuickMutation mutagenesis kit (Beyotime), with primers designed to replace each of the seven cysteine residues with alanine. Fragment of *HERC2* encoding 2540 to 2700th residues was cloned into pcDNA3.1(−)-myc.

### Protein expression and purification

N-terminal 6 × histidine-tagged NCOA4(383–522nd) or NCOA4 mutants were overexpressed in *E. coli* BL21 (DE3) Rosetta, induced by 0.2 mM IPTG (Beyotime) for 4 h at 37 °C. Cells were lysed by sonication in lysis buffer containing 50 mM NaH_2_PO_4_, 300 mM NaCl, 10 mM imidazole, pH 8. Nickel-nitrilotriacetic acid affinity columns (Smart-Lifesciences Biotechnology Co) were used for his-tagged NCOA4 protein purification.

The human FTH, NCOA4 (383–522nd), and HERC2 (2540–2700th) proteins were expressed as a GST N-terminal fusion protein in *E. coli* BL21 (DE3) Arctic strain. The GST-FTH fusion protein was induced with 0.2 mM IPTG for 16 h at 16 °C. The GST-NCOA4 (383–522nd) and -HERC2 were induced with 0.2 mM IPTG for 4 h at 37 °C. Cells were harvested by centrifugation, resuspended in PBS pH 7.4 (140 mM NaCl, 2.7 mM KCl, 10 mM Na_2_HPO_4_, 1.8 mM KH_2_PO_4_, pH 7.3), and lysed by sonication. The purification approach follows the manufacturer’s guidance (GE Healthcare).

### Fe-S cluster reconstitution and stoichiometric determination

To obtain apo-NCOA4, the purified NCOA4 was incubated with EDTA and potassium ferricyanide in a molar ratio of protein: EDTA: ferricyanide in 1:50:20 at 25 °C and incubated for 20 to 30 min till the extensive loss of color. The solution was dialyzed in buffer (50 mM Tris–HCl, pH 7.5, 200 mM NaCl, and 5 mM DTT) and was used as apo-NCOA4 for spectral scanning.

Purified NCOA4 proteins were brought into a glove box for chemical reconstitution of the Fe-S cluster. The protein was incubated with a 10-fold molar excess of iron ammonium sulfate and Na_2_S to protein concentration at 22 °C for 8 h. The reconstituted protein was then passed through a G25 column to remove unbound iron and sulfate. The reconstituted protein was immediately used for spectral studies and iron and sulfide chemical analysis.

The total iron content of reconstituted-NCOA4 was measured using a colorimetric ferrozine-based assay with some modifications (37, 38). Briefly, freshly purified holo-NCOA4 (50 μl) was heated at 95 °C for 20 min after treatment with 37% HCl (11 μl), then centrifuged at 15,000*g* for 10 min. The supernatant was transferred very gently into fresh tubes. Ascorbate was added to reduce the Fe (III) into Fe (II). After 2 min of incubation at room temperature, 18 μl ferrozine (10 mM) and saturated ammonium acetate were sequentially added to each tube. The concentration of iron was determined by measuring the absorbance of the product, iron–ferene complex, at 562 nm. The value was compared with a standard curve prepared from dilutions of freshly prepared Fe (III) solution in the range of 0 to 500 μM.

The acid-labile sulfide content of reconstituted-NCOA4 was measured using a previously described procedure (39). The freshly prepared Na_2_S solution was used as standard in the range of 0 to 500 μM of S^2−^. The protein sample or standard (200 μl) was mixed with 0.6 ml of zinc acetate (1% w/v), followed by the addition of 50 μl of NaOH (12% w/v). After incubation for 15 min, 0.15 ml of N, N-dimethyl-p-phenylenediamine dihydrochloride (0.1% w/v dissolved in 5 M HCl) and 0.15 ml of freshly prepared 10 mM FeCl_3_ (dissolved in 1 M HCl) was added. The reaction mixture was further incubated for 30 min at room temperature, and the absorbance of the product, methylene blue, was measured at 670 nm. For iron and sulfide stoichiometry estimation, three independent preparations of holo-NCOA4 were analyzed to ensure reproducibility, and three dilutions of each sample were considered.

### UV/vis absorption spectrometry

UV/vis absorption was recorded with 200 μl of protein samples right after elution from nickel-nitrilotriacetic acid beads at room temperature on a Spectrophotometer (Molecular Devices) ranging from 280 to 700 nm. The protein samples were at the concentrations of 1 mg/ml to 8 mg/ml in the buffer containing 50 mM NaH_2_PO_4_, 300 mM NaCl, 250 mM imidazole, pH 8.

### EPR spectroscopy

X-band (∼9.6 GHz) EPR spectra were recorded using EMX plus 10/12 spectrometer (Bruker), equipped with Oxford ESR-910 liquid helium cryostat. Briefly, 1 mM oxidized NCOA4 (as-isolated NCOA4) in Tris buffer (50 mM Tris, pH 8) and 10% (v/v) glycerol were transferred into a 4 mm diameter quartz EPR tube (Wilmad 707-SQ-250 M) and frozen in liquid nitrogen. EPR signals of oxidized NCOA4 were recorded at various temperatures (10 K, 25 K, and 45 K). Parameters for recording the EPR spectra were typically 2 G modulation amplitude, 9.40 GHz microwave frequency, and 2 mW incident microwave power; sweep time was 64 s.

### Native mass spectrometry

NCOA4(383–522nd) protein was buffer exchanged into aqueous Tris buffer (10 mM, pH 8) using a micro bio-spin chromatography column (Bio-Rad) and a centrifuge operated at 4 °C. Data were acquired using a Waters Xevo G2-QTOF mass spectrometer (Waters Co). Mass spectra were analyzed using MassLynx v4.1 (https://www.waters.com, Waters Co).

### Preparation of ^57^FeCl_3_ and samples for Mössbauer spectroscopy

^57^Fe (96% enrichment, 10 mg) was converted into iron chloride by dissolving in 0.75 ml of concentrated (36%) hydrochloric acid at 80 °C. This stock solution was added directly to the cultivation medium.

*E. coli* BL21 (DE3)-Rosetta containing plasmid-encoded NCOA4 (383–522nd) hosted by the vector pQE80L and a reference strain with empty pQE80L vector as the negative control were grown in LB medium. After the optical density at 600 nm reached 0.5, 40 μM ^57^FeCl_3_ and 200 μM IPTG were added for protein induction. After 4 h, cells were harvested, washed, and centrifuged. Approximately 600 mg of cell pellets were transferred to a Mössbauer cup made of polyetheretherketone and frozen in liquid nitrogen.

### ^57^Fe Mössbauer spectroscopy

Mössbauer spectra were recorded on a conventional spectrometer with alternating constant acceleration of the γ-source (57Co/Rh, 0.925 GBq), which was kept at room temperature. The minimum experimental line width was 0.24 mm/s (full width at half-height). The sample temperature was maintained constantly low in a liquid nitrogen Mössbauer cryostat produced by Cryo Industries of American Inc. Isomer shifts are quoted relative to iron metal at 300 K. The zero-field spectra were simulated with Lorentzian doublets with the program mf.SL developed by Dr Eckhard Bill at the MPI CEC.

### Immunoprecipitation and immunoblotting

HEK293T/HEK293 cells were transiently transfected with the indicated plasmids using the Hieff Trans Liposomal Transfection Reagent (Yeasen Biotechnology). Post transfection 24 h, cells were harvested and lysed in lysis buffer (50 mM Tris-Cl pH 7.5, 150 mM NaCl, 0.1% NP-40), supplemented with protease inhibitors (Complete ULTRA, Roche). Immunoprecipitations of HA-tagged proteins were carried out using anti-HA magnetic beads (MCE) for 4 h at 4 °C. The beads were then washed three times in a lysis buffer. Immunoprecipitants were separated by SDS-PAGE and transferred to either 0.45 mm nitrocellulose membrane for Western blotting. The information for primary antibodies is as follows: anti-NCOA4 (cat#ab86707) from Abcam, anti-HA (cat#14832901) from Covance, anti-His (cat#A00186), and anti-myc (cat#A00704) from Genscript, anti-FXN, ferritin light chain, ISCU, FTH, and IRP2 (polyclonal, self-made, and raised from rabbits). All self-made antibodies were validated in previous studies (35, 36, 38).

### Aconitase activity assays

In-gel aconitase activity assays were performed as described previously (40). Briefly, aconitase activity gels are composed of a separating gel containing 8% acrylamide and a stacking gel containing 4% acrylamide. The running buffer contains 25 mM Tris pH 8.3, 192 mM glycine, and 3.6 mM citrate. The sample buffer contains 25 mM Tris-Cl, pH 8, 10% glycerol, and 0.025% bromophenol blue. Electrophoresis was carried out at 100 V at 4 °C. Aconitase activities were assayed by incubating the gel in the dark at 37 °C in 100 mM Tris (pH 8), 1 mM NADP, 2.5 mM *cis-*aconitic acid, 5 mM MgCl_2_, 1.2 mM thiazolyl blue tetrazolium bromide, 0.3 mM phenazine methosulfate, and isocitrate dehydrogenase.

### Determination of intracellular Fe^2+^ levels

HEK293 cells were seeded in a 12-well plate. After treatment, cells were washed three times in Hanks' balanced salt solution. Then, the cells were stained with 1 μM FerroOrange in Hanks' balanced salt solution for 30 min at 37 °C and imaged immediately with a fluorescence microscope (Invitrogen EVOS M5000).

### Statistical analysis

All data were graphed and analyzed with GraphPad Prism 8.0 (https://www.graphpad.com/). All data are presented as mean ± SD. Statistical analyses were performed using Student’s *t* test or one-way ANOVA was performed. Differences with a *p* < 0.05 were considered significant.

## Data availability

All data described in this study are presented in the article and accompanying supporting information.

## Supporting information

This article contains supporting information.

## Conflict of interest

The authors declare that they have no conflicts of interest with the contents of this article.
