# Supplementary material for: NCOA4 requires a [3Fe-4S] to sense and maintain the iron homeostasis
Source: J Biol Chem. 2023 Dec 28;300(2):105612. doi: 10.1016/j.jbc.2023.105612 (PMC10831263; doi:10.1016/j.jbc.2023.105612)

**Supplemental information**

**NCOA4 requires a [3Fe-4S] to sense and maintain the iron homeostasis**

Hongting Zhao, Yao Lu, Jinghua Zhang, Zichen Sun, Chen Cheng, Yutong Liu, Lin Wu, Meng Zhang, Weijiang He, Shuangying Hao, Kuanyu Li

**Supplemental figure 1**


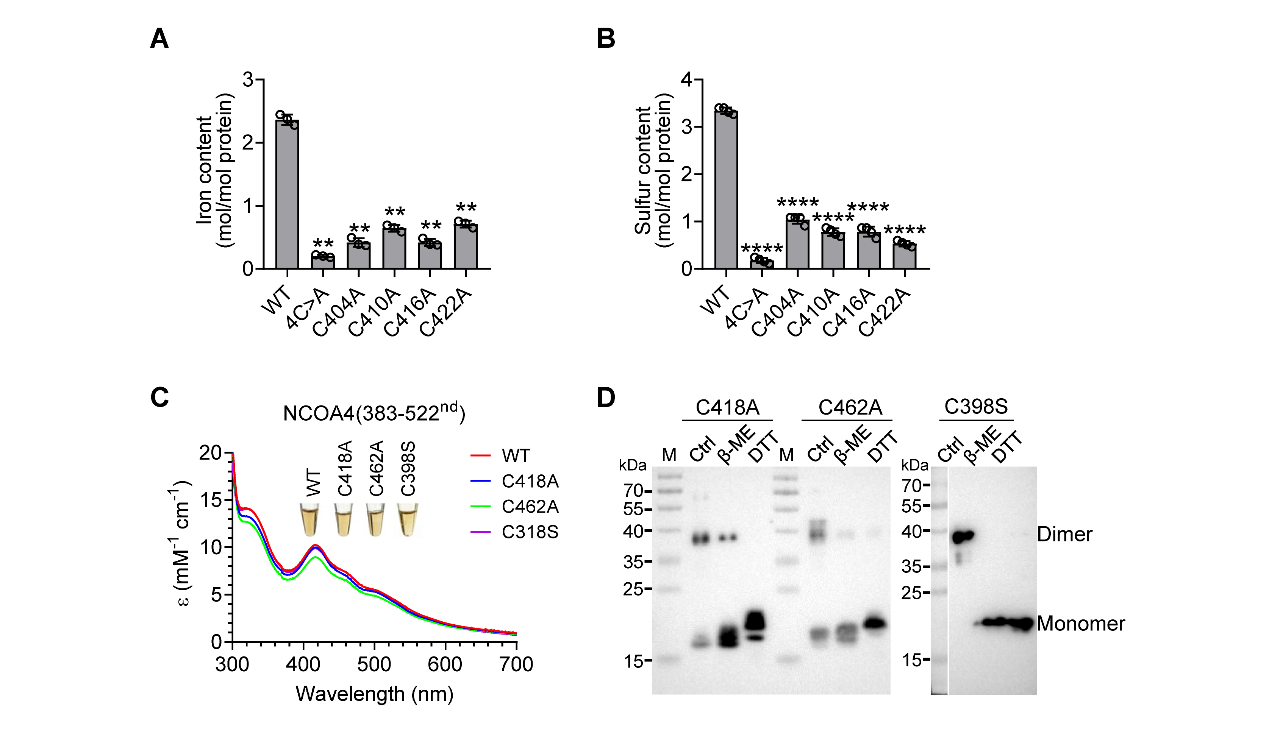


**Fig. S1 Four predicted coordinated cysteines mutation abolishes the Fe-S cluster docking.** (A) The iron content in the purified NCOA4(383-522^nd^) WT and mutants, detected by ferrozine assays. (B) The inorganic sulfur content in the purified NCOA4(383-522^nd^) WT and mutants, detected by methylene blue assays. (C) Colors and the normalized UV/vis absorption spectra of the purified NCOA4(383-522^nd^) mutants C418A, C462A, and C398S. (D) Non-reducing gel electrophoresis analysis of mutants treated with β-ME (10 mM) or DTT (5 mM).

**Supplemental figure 2**


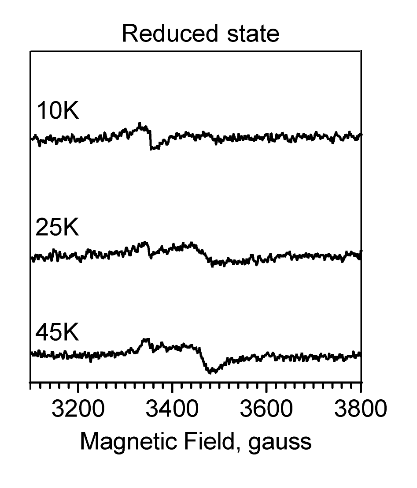


**Fig. S2 Continuous-wave EPR spectra recorded on NCOA4(383-522^nd^) in a reduced state.** The EPR signal of purified NCOA4(383-522^nd^) was recorded following dithionite addition at different temperatures.

**Supplemental figure 3**


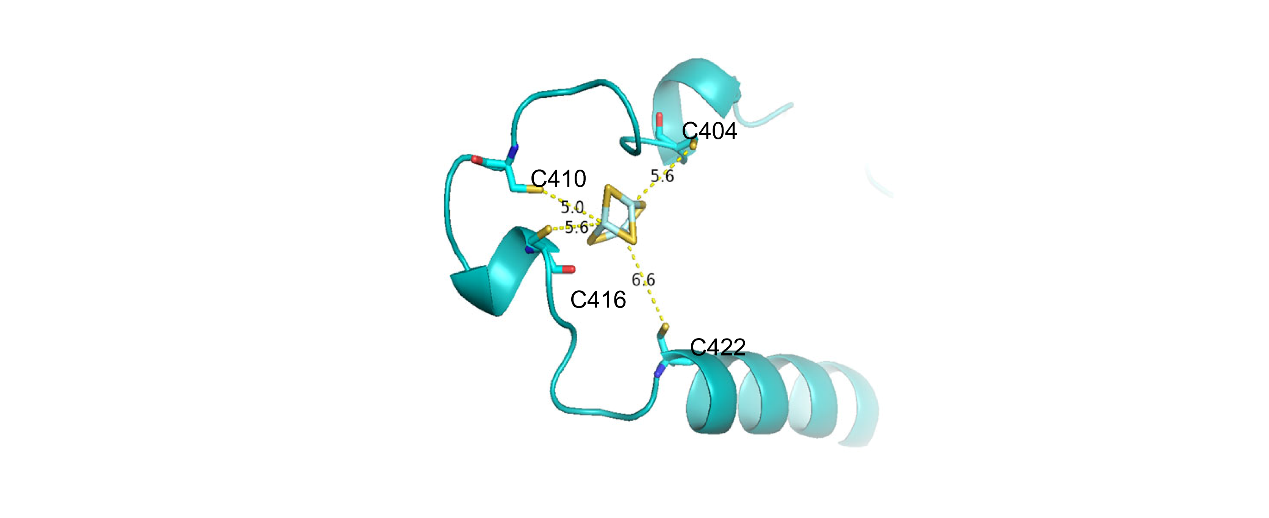


**Fig. S3 [3Fe-4S]-docking with NCOA4 using the Autodock software (version 4.2.6, The Scripps Research Institute).** The structure of the human NCOA4 was retrieved from Alphafold (AF-Q13772-F1). Explicit hydrogen atoms were added, and all water molecules were then deleted. The protein structure was processed using AutoDock Tools as setting Grid box size of 50/22/44 around centre 9.803/43.855/3.926. Ligand [3Fe-4S] were prepared for docking by using AutoDock Tools to assign AD4 atom types and calculate Gasteiger charges. The exhaustiveness parameter was set as default. The closest distances between iron to cysteinyl sulfhydryl are labeled (5.0-6.6 Å).

**Table S1. Plasmids used in this study**


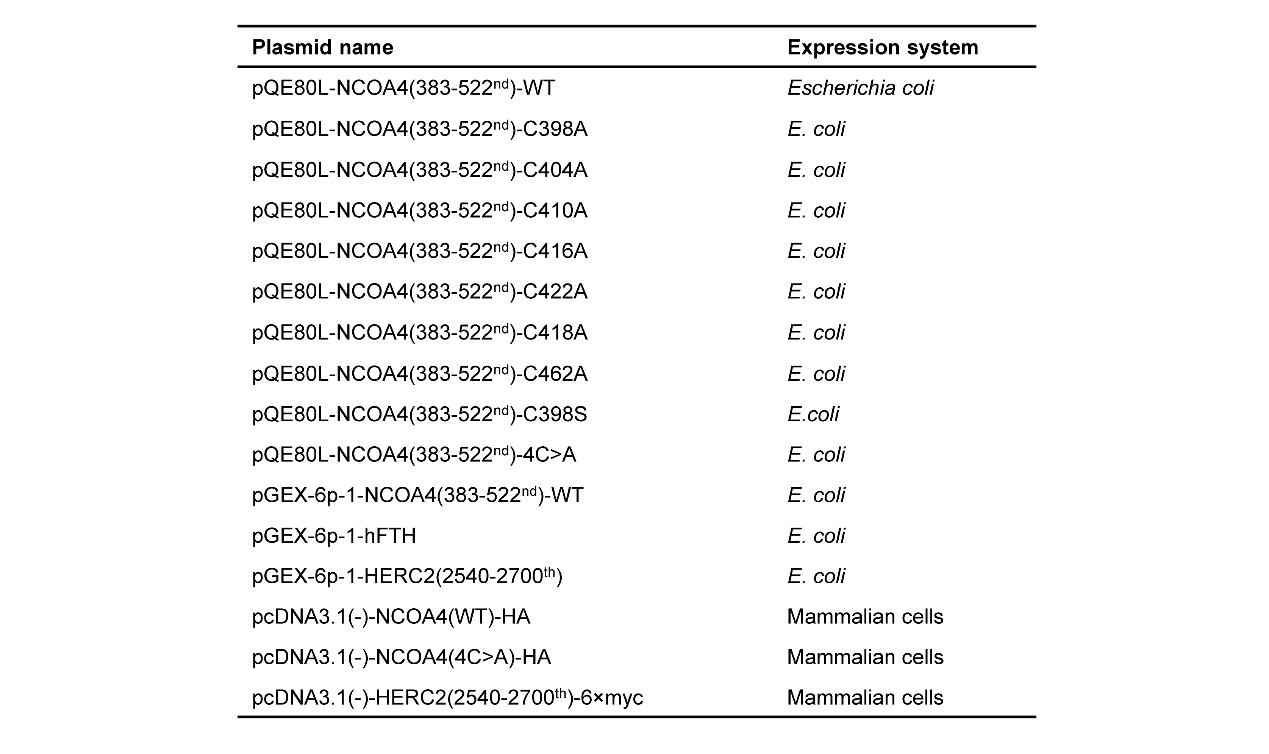

Supplement: Supplemental Figure 1–3 and Table S1 [file mmc1.docx]
